# Effect of Gegen Qinlian Decoction on Hepatic Gluconeogenesis in ZDF Rats with Type 2 Diabetes Mellitus Based on the Farnesol *X* Receptor/Ceramide Signaling Pathway Regulating Mitochondrial Metabolism and Endoplasmic Reticulum Stress

**DOI:** 10.1155/2021/9922292

**Published:** 2021-08-10

**Authors:** Qi Zhou, Ning Song, Shi-qi Wang, Yan Wang, Yi-kun Zhao, Xiang-dong Zhu

**Affiliations:** ^1^Gansu University of Chinese Medicine, Lanzhou, Gansu 730000, China; ^2^Third People's Hospital of Gansu Province, Lanzhou, Gansu 730000, China

## Abstract

**Background:**

Type 2 diabetes mellitus (T2DM) is a kind of disorder of glucose and lipid metabolism with the main clinical manifestation of long‐term higher blood glucose level than the normal value. Farnesol *X* receptor (FXR)/ceramide signaling pathway plays an important role in regulating cholesterol metabolism, lipid homeostasis, and the absorption of fat and vitamins in diet. Gegen Qinlian Decoction (GQD) is a classical herbal formula, which has a good clinical therapeutic effect on diabetes-related metabolic syndrome.

**Objective:**

To investigate the effect of Gegen Qinlian Decoction (GQD) on hepatic gluconeogenesis in obese T2DM rats based on the FXR/ceramide signaling pathway regulating mitochondrial metabolism and endoplasmic reticulum stress (ERS).

**Methods:**

ZDF (fa/fa) rats were fed with high-fat diet to establish the T2DM model; GQD was given to T2DM model rats by gavage; changes of the general state and body weight of rats were recorded; fasting blood glucose was detected; blood insulin, blood ceramide, glycosylated hemoglobin in blood, acetyl CoA in liver mitochondria, and bile salt lyase in intestinal tissue were detected by ELISA. The content of T-*β*-MCA in blood was detected by LC-MS; the content of glycogen in liver tissue was detected by PAS staining; the expression of FXR, Sptlc2, and Smpd3 in ileum tissue, P-PERK, ATF6*α*, GRP78 BIP, and P-IRE1 in the liver, and CS and PC protein in liver mitochondria was detected by immunohistochemistry and western blot assay. The mRNA expression levels of FXR, Sptlc2, and Smpd3 in the ileum, PERK, ATF6*α*, GRP78 BIP, and IRE1 in the liver, and CS and PC in liver mitochondria were detected by qRT-PCR.

**Results:**

GQD can improve the general state of T2DM rats, slow down their weight gain, reduce the levels of fasting blood glucose, fasting insulin, glycosylated hemoglobin, blood ceramide, bile salt hydrolase in intestinal tissue, and acetyl CoA in liver mitochondria of T2DM rats, and increase the contents of liver glycogen and T-*β*-MCA in blood of T2DM rats. At the molecular level, GQD can inhibit the expression levels of FXR, Sptlc2, and Smpd3 in the ileum of T2DM rats and the protein and mRNA expression levels of oxidative stress-related factors in the liver. At the same time, GQD can increase the expression of CS and reduce the expression of PC in liver mitochondria of T2DM rats.

**Conclusion:**

GQD can inhibit the FXR/ceramide signaling pathway, regulate endoplasmic reticulum stress, enhance the CS activity of liver mitochondria, reduce the acetyl CoA level and PC activity of liver mitochondria, inhibit hepatic gluconeogenesis, protect islet *β*-cells, and control blood glucose.

## 1. Introduction

Type 2 diabetes mellitus (T2DM) is a kind of disorder of glucose and lipid metabolism with the main clinical manifestation of long‐term higher blood glucose level than the normal value [1]. The disorder of gluconeogenesis and glycogen decomposition leads to the increase of liver glucose output, which is the main pathological factor for the increase of fasting blood glucose in patients with T2DM [2–4]. Therefore, inhibiting excessive hepatic gluconeogenesis and reducing endogenous glucose production is one of the main targets of clinical drug therapy for T2DM [3, 5].

Gegen Qinlian Decoction (GQD) is a classical herbal formula, which was firstly recorded in the Treatise on Exogenous Febrile Disease of the Han Dynasty. It is composed of Gegen (Puerariae Lobatae Radix), Huanglian (Coptidis Rhizoma), Huangqin (Scutellariae Radix), Gancao (Glycyrrhizae Radix et Rhizoma), and Jiang (*Zingiber officinale* Rosc.). 12 pharmacodynamic components were determined by HPLC [6, 7] as follows: puerarin, baicalin, glycyrrhizin, daidzein, ammonium glycyrrhizinate, berberine hydrochloride, wogonin, berberine hydrochloride, jatrorrhizine hydrochloride and salt acid epiberberine, palmatine hydrochloride, baicalein, and wogonin. Among them, puerarin, baicalin, baicalein, wogonin, berberine, and ammonium glycyrrhizinate could improve insulin resistance and also have good therapeutic effects on cardiovascular disease, diabetic nephropathy, diabetic retinopathy, and other complications caused by diabetes [8–17]. In animal experiments, studies have confirmed that GQD can play a role in the treatment of T2DM from regulating glucose and lipid metabolism, reducing oxidative stress response, changing the structure of intestinal flora, reducing the expression levels of inflammatory factors, and improving insulin resistance [7, 18, 19]. However, further elucidation of its possible molecular mechanism is still of great significance for the discovery and clinical application of monomer drugs of GQD.

Farnesol *X* receptor (FXR) is a bile acid receptor, which plays an important role in regulating cholesterol metabolism, lipid homeostasis, and the absorption of fat and vitamins in diet by regulating the expression of target genes, so it is an important drug target for metabolic diseases [20–22]. In recent years, studies have found that FXR plays an important role in human glucose and lipid metabolism, and its abnormal activation can promote fatty liver, T2DM, dyslipidemia, obesity, and other diseases [23–25]. Tauro-*β*-muricholic acid (T-*β*-MCA) is a natural FXR antagonist in mice [26, 27]. Increasing intestinal T-*β*-MCA level can improve hyperlipidemia induced obesity, impaired glucose tolerance, and hepatic steatosis by inhibiting intestinal FXR signaling pathway. However, T-*β*-MCA is rapidly hydrolyzed to *β*-MCA by bile salt hydrolase (BSH) secreted by intestinal flora [28]. Therefore, inhibition of BSH can block intestinal FXR signal transduction by increasing intestinal T-*β*-MCA [27]. Further studies showed that FXR could regulate the expression of sphingomyelin phosphodiesterase 3 (Smpd3) and serine palmitoyl transferase, long-chain base subunit 2 (Sptlc2) by binding to the upstream FXR binding sites of their genes [28]. Ceramide can further inhibit mitochondrial glucose metabolism, lead to endoplasmic reticulum stress (ER) in liver tissue, damage islet *β* cells, and promote the development of T2DM.

Therefore, this study will explore the therapeutic effect and the related molecular mechanism of GQD on T2DM rats for the first time based on FXR-ceramide signaling pathway, so as to provide guidance for clinical application of GQD.

## 2. Materials and Methods

### 2.1. Materials

Thirty-two ZDF (fa/fa) rats and eight ZDF (fa/+) rats were purchased from Beijing Viton Lihua Experimental Animal Technology Co., Ltd. The production license number is SCXK (Beijing) 2016–0006.

CAPE (BSH inhibitor) (Dalian Meilun, M0721A); glycosylated hemoglobin (HbA1c) assay kit (Nanjing Jiancheng, A056-1-1); Carnoy's solution (Solarbio, G2312); glycogen periodic acid schiff (PAS/Hematoxylin) Stain Kit (Solarbio, G1281); hematoxylin-eosin (HE) staining kit (Solarbio, G1120); immunohistochemistry kit (ZSGB-BIO, SP9001); insulin Elisa kit (mlbio, ml302840); ceramide Elisa kit (mlbio, ml059168); acetyl coenzyme A Elisa kit (mlbio, ml110767-c); bile salt hydrolase (BSH) Elisa kit (Solarbio, ml602113-c); RIPA lysate (Solarbio, R0010); mitochondrial extraction kit (Solarbio, SM0020); total RNA extraction kit (Takara, 9109); PrimeScript™ RT reagent Kit with gDNA Eraser (Perfect Real Time) (RR047A, Takara); TB Green® Premix Ex Taq™ II (Tli RNaseH Plus) (Takara, RR820L); SDS-PAGE Gel kit (Solarbio, P1200); rabbit anti-P-PERK polyclonal antibody (ImmunoWay, YP1055); rabbit anti-ATF6 alpha polyclonal antibody (Abcam, ab203119); rabbit anti-GRP78 BIP polyclonal antibody (Abcam, ab21685); rabbit anti-P-IRE1 polyclonal antibody (Abcam, ab48187); rabbit anti-FXR polyclonal antibody (Invitrogen, PA5-40755); mice anti-smpd3 monoclonal antibody (Santa Cruz, sc-166637); rabbit anti-Sptlc2 polyclonal antibody (Abcam, ab23696); rabbit anti-PC polyclonal antibody (Abcam, ab229267); and rabbit anti-CS polyclonal antibody (Abcam, ab96600).

### 2.2. Methods

#### 2.2.1. Preparation of GQD

According to the ratio of 3 : 3 : 3 : 2 : 0.5 w/w/w/w/w, Gegen (Puerariae Lobatae Radix), Huangqin (Scutellariae Radix), Huanglian (Coptidis Rhizoma), Gancao (Glycyrrhizae Radix et Rhizoma), and Jiang (*Zingiber officinale* Rosc.) were weighed and soaked in 8 times of distilled water for 30 minutes. The Gegen was decocted for 30 minutes, and then the remaining drugs were decocted for 30 minutes together. The GQD extracts were filtered by screen. The residue was decocted with 8 times of distilled water for 30 minutes. The GQD extracts were filtered by screen again and combined twice.

#### 2.2.2. Animal Modeling and Group Intervention

After 3 weeks of feeding with LabDiet 5C08 high-fat diet (HFD), 32 male ZDF (fa/fa) rats with random blood glucose ≥ 16.7 mmol/l on different two days were randomly divided into 4 groups with 8 rats in each group: model group (model), CAPE group (CAPE), high-dose Gegen Qinlian Decoction group (high-dose GQD), low-dose Gegen Qinlian Decoction group (low-dose GQD), and ZDF (fa/+) rats as blank group (blank). The model group and blank group were given 10 ml/kg normal saline by gavage; the high-dose GQD group was given 14.3 g/kg GQD by gavage; the low-dose GQD group was given 3.1 g/kg GQD by gavage; and the CAPE group was given 50 mg/kg CAPE by gavage. All animals were treated by gavage once a day for 12 weeks.

#### 2.2.3. General Condition and Weight

During the treatment, the frequency of urination, mental state, animal behavior, water intake, and food intake of rats were observed, and the rat body weight was recorded once a week.

#### 2.2.4. Detection of Fasting Plasma Glucose (FPG)

FPG levels were measured by blood glucose meter once a week.

#### 2.2.5. Blood Sample Collection

After 12 weeks of treatment, the rats were anesthetized with 3% pentobarbital sodium; the blood was collected from inferior vena cava and stored in EDTA anticoagulant tube and ordinary blood collection vessel, respectively. After part of the whole blood was retained in EDTA anticoagulant tube, the remaining blood was centrifuged at 3500 r/min for 5 min by low-temperature centrifuge. Plasma and serum were separated and then packed in EP tube and frozen in −80°C ice box for detection of biochemical indicators.

#### 2.2.6. Tissue Specimen Collection

After the animals were euthanized, the ileum, cecum, and liver tissues were quickly separated and washed with 4°C normal saline. Part of the liver tissue was fixed with Carnoy's solution for PAS staining; another part of the liver tissue was fixed in 4% paraformaldehyde solution for paraffin embedding for immunohistochemistry. The remaining liver, ileum, and cecum tissues were rinsed with normal saline and immediately frozen in liquid nitrogen for the detection of biochemical indicators.

#### 2.2.7. The Concentrations of Blood Insulin, Blood Ceramide, Blood Glycosylated Hemoglobin, Acetyl-Coenzyme A in Liver Mitochondria, and BSH in Intestinal Tissue Were Detected by ELISA

The concentrations of insulin, ceramide, glycosylated hemoglobin, acetyl-coenzyme A, and bile salt hydrolase were detected by ELISA, and the specific steps were in accordance with the instructions of the kit.

#### 2.2.8. T-*β*-MCA Content Detection

The content of T-*β*-MCA in blood was detected by LC-MS.

#### 2.2.9. The Content of Glycogen in Liver Tissue Was Detected by PAS Staining

The liver tissue was fixed and embedded in paraffin and then made into 5 *μ*m thick sections. After washing with distilled water, the sections were stained with periodate acid solution, Schiff's solution, and Halley's hematoxylin and then dehydrated, cleared, and coverslipped. The sections were observed with light microscope.

#### 2.2.10. Immunohistochemistry Was Used to Detect the Expression Levels of FXR, Sptlc2, and Smpd3 in the Ileum, P-PERK, ATF6*α*, GRP78 BiP, and P-IRE1 in the Liver Endoplasmic Reticulum, and CS and PC in the Liver Mitochondria

The roasted tissue sections were dewaxed, hydrated, and then retrieved by citric acid buffer (PH6.0) microwave antigen retrieval, incubated with 3% hydrogen peroxide at room temperature for 10 min, and goat serum was sealed at room temperature for 15 min. The sections were incubated overnight at 4°C with primary antibodies diluted in PBS. After being washed with PBS, the tissue sections were incubated at 37°C for 30 min with the biotin labeled secondary antibody in SP kit. The HRP-labeled *Streptomyces* ovalbumin working solution was added to the section and incubated at 37°C for 10 min. The sections were washed with PBS and developed with DAB at room temperature and then stained with hematoxylin. After dehydration, the slices were cleared with xylene and mounted with neutral gum. After drying, the slices were preserved and photographed for analysis.

#### 2.2.11. Western Blot Was Used to Detect the Expression of FXR, Sptlc2, and Smpd3 in the Ileum, p-PERK, ATF6*α*, GRP78 BiP, and p-IRE1 in the Liver Endoplasmic Reticulum, and CS and PC in the Liver Mitochondria

The ileum tissues used to detect FXR, Sptlc2, and Smpd3 proteins and the liver tissues used to detect p-PERK, ATF6*α*, GRP78 BIP and p-IRE1 proteins were directly lysed with RIPA lysate containing 1 mM PMSF. The liver mitochondria used to detect CS and PC proteins were firstly extracted with mitochondrial extraction kit, and then the proteins in mitochondria were extracted with RIPA lysate (1 mM PMSF). After the BCA kit quantified the protein content in the lysate, 4x loading buffer was used to denature the protein samples. The proteins were separated by SDS-PAGE gel electrophoresis. After being transferred from the gel to the membrane, the membrane was blocked for 1 h with 2.5% bovine serum albumin, and then incubated with appropriate dilutions of primary antibody in blocking buffer overnight at 4°C, washed with TBST and incubated with the dilution of conjugated secondary antibody in blocking buffer for 2 h at room temperature. After washing with TBST, the ECL luminescent solution was added and exposed by gel imager. The gray values of the bands were analyzed by ImagePro Plus (IPP), and semiquantitative analysis was carried out.

#### 2.2.12. qRT-PCR Was Used to Detect the Expression of *FXR, Sptlc2,* and *Smpd3* in the Ileum, *PERK, ATF6α, GRP78 BiP,* and *IRE1* in the Liver Endoplasmic Reticulum, and *CS* and *PC* mRNA in the Liver Mitochondria

The total mRNA of the ileum and liver tissue was extracted with TRIzol reagent. The amplification procedure was as follows: predenaturation: 95°C, 5 min, 1 cycle; amplification: 95°C, 10 s, 60°C, 30 s, 40 cycles. The primer sequences are shown in Table 1. 2^−△△CT^ was used to calculate the relative expression of each mRNA.

#### 2.2.13. Statistical Methods

SPSS 22.0 statistical software was used to analyse the measurement data. The measurement data was expressed as mean ± standard deviation ($\bar{x}$ ± s). If the data obeys normal distribution, one-way ANOVA was used for comparison among multiple groups, and LSD was used for pairwise comparison. If the distribution is not normal, the median and interquartile distance are used.

## 3. Results

### 3.1. Effects of GQD on the General Condition of ZDF (fa/fa) Rats

After 12 weeks of intervention, GQD can significantly improve the general condition of ZDF (fa/fa) rats, ZDF (fa/fa) rat activity ability, and mental state are increased with the increase of intervention time, urination, drinking water, and diet are gradually reduced with the increase of intervention time.

### 3.2. Effects of GQD on the Body Weight, Fasting Blood Glucose, Glycosylated Hemoglobin, and Insulin in ZDF (fa/fa) Rats

With the increase of intervention time, the weight of each group of rats gradually increased, but from 1 week after the intervention, the weight gain of rats in the high-, low-dose GQD group and CAPE group was lower than that of rats in the model group, and the weight gain gradually leveled off and gradually approached the weight of rats in the blank group.

With the increase of intervention time, the fasting plasma glucose of rats in the blank group and the model group was always at normal and high levels with no change significantly, respectively. With the increase of intervention time, the fasting plasma glucose levels of rats in the high-, low-dose GQD group and CAPE group gradually decreased.

**Figure 1:**
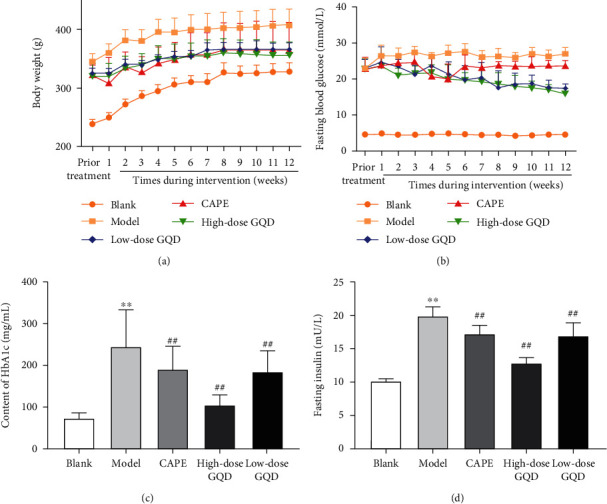
Effects of Gegen Qinlian Decoction (GQD) on the body weight, fasting blood glucose, glycosylated hemoglobin, and insulin in ZDF (fa/fa) rats. (a) Body weight. (b) Fasting blood glucose levels. (c) HbA1c levels. (d) Fasting insulin levels. Results were expressed as mean ± SD (*n* = 8). ^*∗∗*^*P* < 0.01 versus the blank group; ^##^*P* < 0.01 versus the model group.

After 12 weeks of intervention, compared with that of the blank group, the levels of glycosylated hemoglobin and fasting insulin in the blood of the model group rats were significantly increased (*P* < 0.05), compared with those of the model group, the levels of glycosylated hemoglobin and fasting insulin in the blood of the high-dose, low-dose GQD group and CAPE group rats were significantly decreased (*P* < 0.05) (Figure 1).

### 3.3. Effects of GQD on the Glycogen Content of Liver Tissue in ZDF (fa/fa) Rats

**Figure 2:**
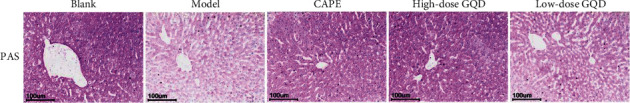
Effect of Gegen Qinlian Decoction (GQD) on the glycogen content of liver tissue in ZDF (fa/fa) rats.

After 12 weeks of intervention, compared with that of rats in the blank group, the liver glycogen content of rats in the model group was significantly reduced; compared with that of rats in the model group, the liver glycogen content of rats in the high-dose, low-dose GQD group, and CAPE group rats was significantly increased (Figure 2).

### 3.4. Effects of GQD on the Levels of Ceramide in Blood of ZDF (fa/fa) Rats

**Figure 3:**
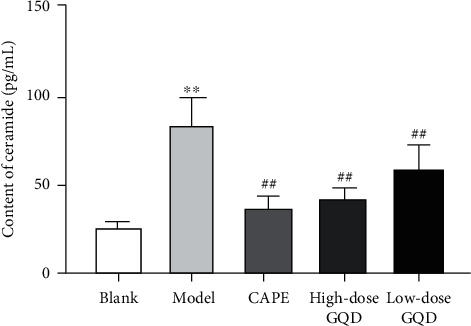
Effects of Gegen Qinlian Decoction (GQD) on the levels of ceramide in blood of ZDF (fa/fa) rats. Results were presented as mean ± SD (*n* = 8). ^*∗∗*^*P* < 0.01 versus the blank group; ^##^*P* < 0.01 versus the model group.

After 12 weeks of intervention, compared with those of rats in the blank group, the levels of the ceramide in the blood of the model group rats were significantly increased (*P* < 0.05); compared with those of rats in the model group, the levels of the ceramide in the blood of the high-dose, low-dose GQD group, and CAPE group rats were significantly decreased (*P* < 0.05) (Figure 3).

### 3.5. Effects of GQD on the Content of Tauro-*β*-Muricholic Acid (T-*β*-MCA) in Blood of ZDF (fa/fa) Rats

**Figure 4:**
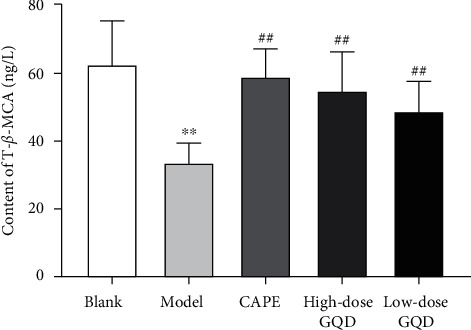
Effect of Gegen Qinlian Decoction (GQD) on the content of tauro-*β*-muricholic acid (T-*β*-MCA) in blood of ZDF (fa/fa) rats. Results were presented as mean ± SD (*n* = 8). ^*∗∗*^*P* < 0.01 versus the blank group; ^##^*P* < 0.01 versus the model group.

After 12 weeks of intervention, compared with those of rats in the blank group, the levels of the T-*β*-MCA in the blood of the model group rats were significantly decreased (*P* < 0.05); compared with those of rats in the model group, the levels of the T-*β*-MCA in the blood of the high-dose, low-dose GQD group and CAPE group rats were significantly increased (*P* < 0.05) (Figure 4).

### 3.6. Effects of GQD on the Content of Bile Salt Hydrolase (BSH) in Cecum Tissue of ZDF (fa/fa) Rats

**Figure 5:**
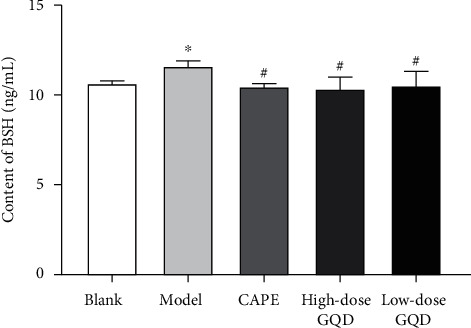
Effect of Gegen Qinlian Decoction (GQD) on the content of bile salt hydrolase (BSH) in the cecum of ZDF (fa/fa) rats. Results were presented as mean ± SD (*n* = 8). ^*∗∗*^*P* < 0.01 versus the blank group; ^##^*P* < 0.01 versus the model group.

After 12 weeks of intervention, compared with those of rats in the blank group, the levels of the BSH in the cecum tissue of the model group rats were significantly increased (*P* < 0.05); compared with those of rats in the model group, the levels of the BSH in the cecum tissue of the high-dose, low-dose GQD group and CAPE group rats were significantly decreased (*P* < 0.05) (Figure 5).

### 3.7. Effects of GQD on FXR, Sptlc2, and Smpd3 Expressions in Intestinal Tissue of ZDF (fa/fa) Rats

**Figure 6:**
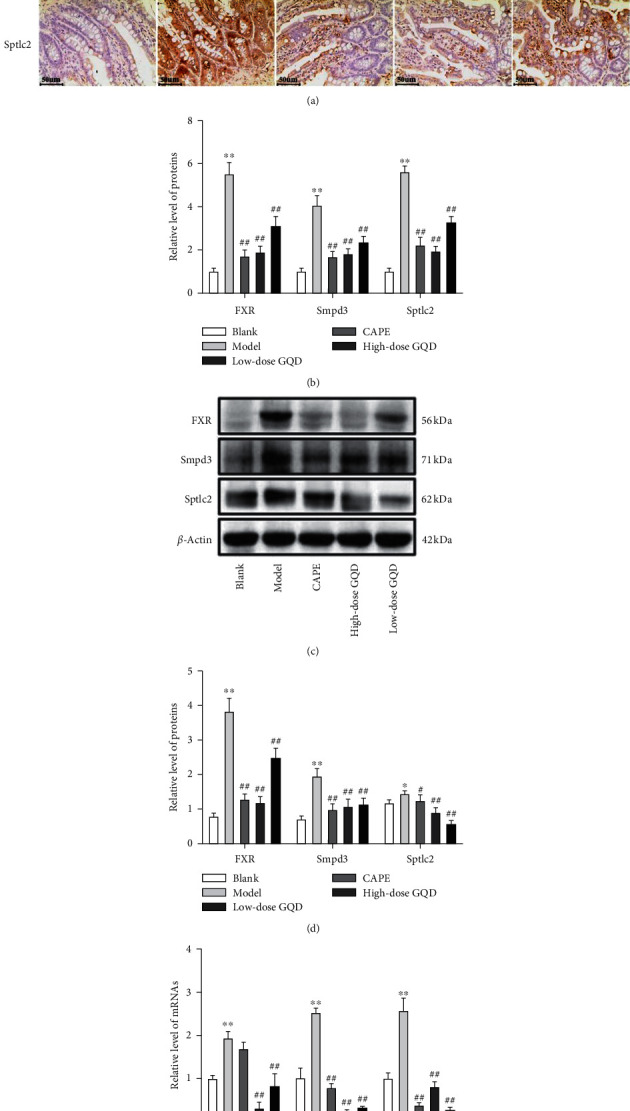
Effects of the treatment of Gegen Qinlian Decoction (GQD) for 12 weeks on FXR, Sptlc2, and Smpd3 expressions in the intestinal tissue of ZDF (fa/fa) rats. The levels of FXR, Sptlc2, and Smpd3 proteins were detected by the immunohistochemistry assay ((a), (b)) and western blot assay ((c), (d)). The levels of FXR, Sptlc2, and Smpd3 mRNAs were detected by the qRT-PCR assay (e). Results were presented as mean ± SD (*n* = 8). ^*∗∗*^*P* < 0.01 versus the blank group; ^##^*P* < 0.01 versus the model group (magnification ×200).

After 12 weeks of intervention, at the protein and mRNA levels, compared with those of rats in the blank group, the levels of the FXR, Sptlc2, and Smpd3 expressions in the intestinal tissue of the model group rats were significantly increased (*P* < 0.05); compared with those of rats in the model group, the levels of the FXR, Sptlc2, and Smpd3 expressions in the intestinal tissue of the high-dose, low-dose GQD group and CAPE group rats were significantly decreased (*P* < 0.05) (Figure 6).

### 3.8. Effects of GQD on the Acetyl-CoA Content in the Mitochondria of Liver Tissue in ZDF (fa/fa) Rats

**Figure 7:**
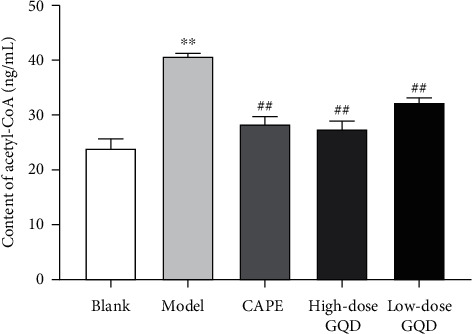
Effects of Gegen Qinlian Decoction (GQD) on the acetyl-coenzyme A content in the mitochondria of liver tissue in ZDF (fa/fa) rats. Results were presented as mean ± SD (*n* = 8). ^*∗∗*^*P* < 0.01 versus the blank group; ^##^*P* < 0.01 versus the model group.

After 12 weeks of intervention, compared with that of rats in the blank group, the content of acetyl CoA in the liver tissue mitochondria of rats in the model group were significantly increased (*P* < 0.05); compared with that of rats in the model group, the content of acetyl-CoA in the liver tissue mitochondria of rats in the high-dose, low-dose GQD group and CAPE group rats were significantly decreased (*P* < 0.05) (Figure 7).

### 3.9. Effects of GQD on CS and PC Expression in the Mitochondria of Liver Tissue in ZDF (fa/fa) Rats

**Figure 8:**
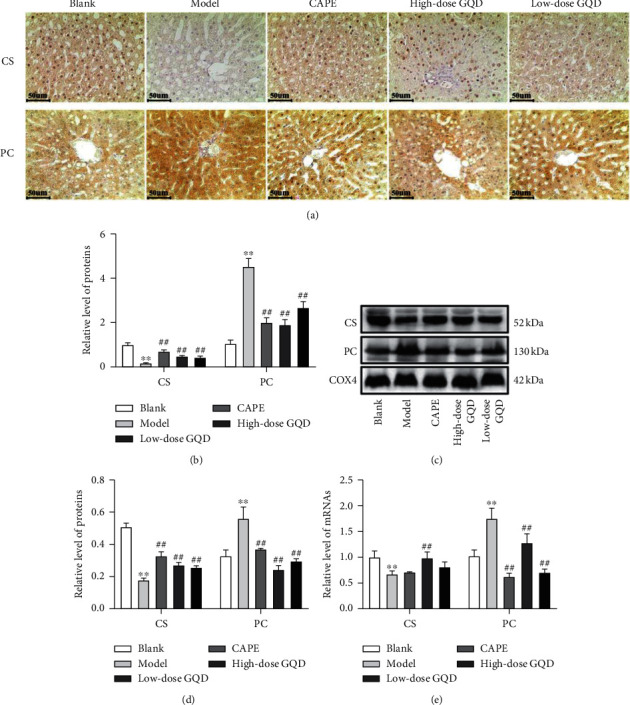
Effects of the treatment of Gegen Qinlian Decoction (GQD) for 12 weeks on CS and PC expressions in the mitochondria of the liver of ZDF (fa/fa) rats. The levels of CS and PC proteins were detected by the immunohistochemistry assay ((a), (b)) and western blot assay ((c), (d)). The levels of CS and PC mRNAs were detected by the qRT-PCR assay (e). Results were presented as mean ± SD (*n* = 8). ^*∗∗*^*P* < 0.01 versus the blank group; ^##^*P* < 0.01 versus the model group (magnification ×200).

After 12 weeks of intervention, at the protein and mRNA levels, compared with those of rats in the blank group, the expression levels of CS in liver mitochondria of the model group rats were significantly decreased (*P* < 0.05), and the expression levels of PC in liver mitochondria of the model group rats were significantly increased (*P* < 0.05); compared with those of rats in the model group, the expression levels of CS in liver mitochondria of the high-dose, low-dose GQD group and CAPE group rats were significantly enhanced (*P* < 0.05), and the expression levels of PC in liver mitochondria of the high-dose, low-dose GQD group and CAPE group rats were significantly decreased (*P* < 0.05) (Figure 8).

### 3.10. Effects of GQD on the Expression of the ER-Related Proteins IRE1, p-IRE1, PERK, p-PERK, ATF6 Alpha, and GRP78 BIP in the Liver of ZDF (fa/fa) Rats

**Figure 9:**
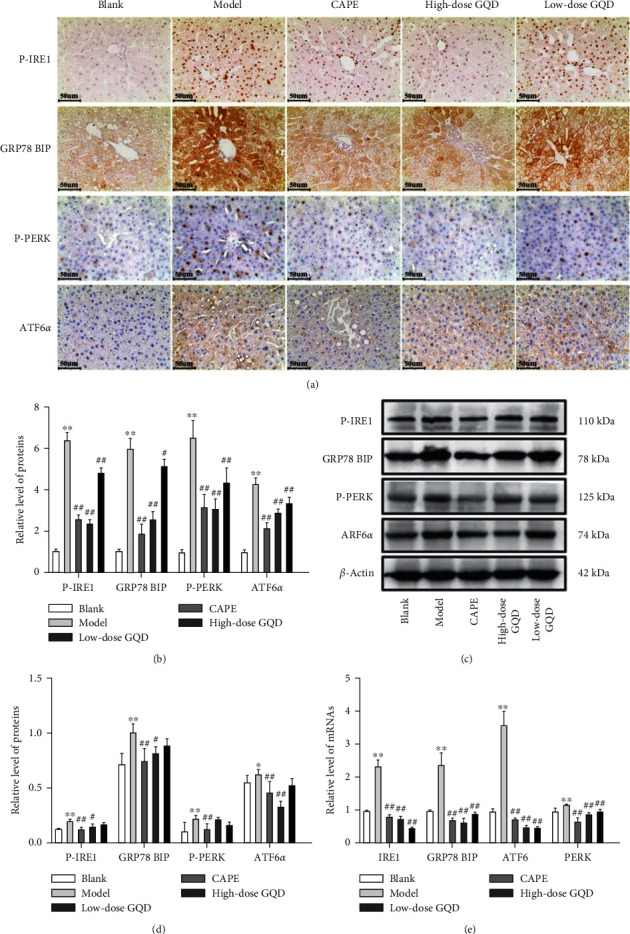
Effects of Gegen Qinlian Decoction (GQD) on the expression of the ER-related proteins IRE1, p-IRE1, PERK, p-PERK, ATF6 alpha, and GRP78 BIP in the liver of ZDF (fa/fa) rats. The levels of p-IRE1, p-PERK, ATF6 alpha, and GRP78 BIP proteins were detected by the immunohistochemistry assay ((a), (b)) and western blot assay ((c), (d)). The levels of IRE1, PERK, ATF6 alpha, and GRP78 BIP mRNAs were detected by the qRT-PCR assay (e). Results were presented as mean ± SD (*n* = 8). ^*∗∗*^*P* < 0.01 versus the blank group; ^##^*P* < 0.01 versus the model group (magnification ×200).

Compared with the blank group, the stress-related proteins p-IRE1, p-PERK, ATF6 alpha, and GRP78 BIP in the model group mice increased significantly (*P* < 0.05), and the corresponding gene mRNA expression also increased significantly (*P* < 0.05). Compared with the model group, p-IRE1, p-PERK, ATF6 alpha, and GRP78 BIP proteins were significantly reduced in the livers of the high-dose groups of CAPE and Gegenyu soup (*P* < 0.05), and the corresponding gene mRNA expression was also significantly reduced (*P* < 0.05) (Figure 9).

## 4. Discussion

This study found that GQD can significantly reduce the frequency of urination and the amount of drinking water and diet of T2DM rats, increase activity, and reduce body weight of T2DM rats. Moreover, GQD can significantly reduce FBG level, serum insulin, and glycosylated hemoglobin content of T2DM rats. In addition, GQD can reduce the ability of gluconeogenesis and promote the synthesis of glycogen in T2DM rats, thus contributing to the reduction of blood glucose. It is suggested above that GQD can improve the diabetic symptoms of T2DM rats, which is almost the same as that of diabetic rats treated with CAPE. Several studies have found that GQD can effectively improve the balance of glucose and lipid metabolism in vitro and in vivo and reduce insulin resistance [7]. Lin et al. found that GQD can effectively improve the symptoms of diabetes and has less side effects through meta-analysis [29], which is consistent with the results of this study.

The molecular mechanisms of GQD in improving the symptoms of T2DM remain unclear. In recent years, the researchers found that the specific inhibition of intestinal FXR can significantly improve the disorder of the glucose and lipid metabolism caused by high-fat diet through a number of different metabolic pathways. Intestinal FXR can be used as a potential important therapeutic target for diabetes. In this study, we found that GQD can reduce the expression of FXR protein and mRNA in the ileum of T2DM rats. At the same time, T-*β*-MCA, as a natural FXR antagonist, increased significantly in obese T2DM rats after GQD intervention, while the level of BSH, which can catalyze the hydrolysis of T-*β*-MCA, decreased significantly. These results suggest that GQD can inhibit the activation and expression of FXR protein in obese T2DM rats and may further inhibit its downstream signal. T-*β*-MCA, as a secondary metabolite of bile acids in the intestine, can be hydrolyzed by BSH secreted by intestinal flora. Therefore, whether GQD can change the production of BSH and the metabolism of T-*β*-MCA and affect the activation of FXR by changing the intestinal flora of obese T2DM rats still needs to be further studied in the future.

The genes of Smpd3 and Sptlc2 proteins, which can catalyze ceramide synthesis in ileum, have FXR binding sites [28]. Intestinal FXR can affect glucose and lipid metabolism through regulating the synthesis of intestinal ceramide [30, 31]. This study showed that GQD could decrease the concentration of ceramide in serum of T2DM rats and inhibit the protein and mRNA levels of Sptlc2 and Smpd3 in ileum. It has been reported that selective inhibition of intestinal FXR can reduce ceramide content in ileum, enhance the activity of liver mitochondrial CS, and then reduce the level of liver mitochondrial acetyl CoA and the activity of liver mitochondrial PC, and then inhibit gluconeogenesis. This study found that GQD could increase the content of CS protein and mRNA in liver mitochondria of T2DM rats; at the same time, the content of acetyl CoA in liver mitochondria of T2DM rats after GQD intervention was significantly reduced, which suggested that GQD could inhibit gluconeogenesis in T2DM rats. As a key metabolic intermediate regulating liver gluconeogenesis in liver, acetyl CoA has a positive regulatory effect on PC activation [32] and independent of hepatic insulin conduction signal [33]. Studies have shown that the specific inhibition of liver PC can prevent obesity, hepatic steatosis, and glucose intolerance caused by high-fat diet (HFD) [34]. This study further showed that GQD could inhibit the content of PC protein and the expression of PC mRNA in the liver of T2DM rats. These results suggest that GQD can reduce the protein expression of Sptlc2 and Smpd3 in the ileum of diabetic rats by inhibiting the expression of FXR in the ileum and then reduce the source of intestinal ceramide in serum, enhance the aerobic oxidation of glucose in liver tissue, enhance glucose metabolism, and inhibit gluconeogenesis, so as to improve the symptoms of T2DM.

Further study showed that the decreased concentration of serum ceramide could regulate the hepatic endoplasmic reticulum stress- (ERS-) related proteins, including PERK, ATF6*α*, CHOP, and GRP78 BIP, and then reduce ER stress induced by high-fat diet and insulin resistance [24, 30, 35]. ER stress is involved in glucose and lipid metabolism [36], and ceramide reduces ER stress to enhance mitochondrial CS activity [30]. This study found that GQD could inhibit the expression of *IRE1*, *PERK*, *ATF6α*, and *GRP78 BIP* mRNA in liver tissue of T2DM rats and inhibit the expression of ATF6*α* and BIP proteins and the phosphorylation of IRE1 and PERK in liver tissue. These factors are the marker proteins of ERS. ERS induced by diabetes can aggravate the damage of islet *β* cells. GQD can reduce ERS in liver tissue of T2DM rats by reducing the content of serum ceramide and then protect islet *β* cells and improve glucose and lipid metabolism.

In conclusion, GQD can inhibit intestinal FXR and reduce the concentration of enterogenous ceramide and serum ceramide. Serum ceramide can regulate liver ERS, enhance liver mitochondrial CS activity, reduce liver mitochondrial acetyl CoA level and PC activity, inhibit liver gluconeogenesis, and protect islet *β* cells, so as to control blood glucose level.

## Figures and Tables

**Table 1:** The primer sequences.

Gene	*F* (5′⟶3′)	*R* (5′⟶3′)
*FXR*	GGACACGCAGACCTGTTGGA	GGATGACAATTGCTGTGAGCAGA
*Sptlc2*	CAGACTGTCGGGAGCAACCA	CTTGTCCGAGGCTGACCATAAAC
*Smpd3*	CTGTGGACTCAGGGCTCGTGTA	GCCTTGTCACGGAATCTGGAA
*PERK*	TTCAATGCGTGGCTGGAAAC	AGGATCCATCTGCCGGATCTTA
*ATF6α*	GAGGCTCAAAGTCCCAAGTCCA	GGCAGGGCTCACACTAGGTTTC
*GRP78 BiP*	TCAGCCCACCGTAACAATCAAG	TCCAGTCAGATCAAATGTACCCAGA
*IRE1*	CATCACCATGTATGACACCAAGACC	TGTCCACAGTTACCACCAGTCCA
*CS*	TGGCCCAACGTAGATGCTCA	AGCCTAGGGCTCTGCTCCAGATA
*PC*	CCTACAGCTGCCACCAAGATGA	CCACAATGCCCGCTAGGAAC
*β-Actin*	GGAGATTACTGCCCTGGCTCCTA	GACTCATCGTACTCCTGCTTGCTG
*COX4*	TGTTGGCTACCAGGGCACTTA	GGTAGTCACGCCGATCAACATA

## Data Availability

The figure and table data used to support the findings of this study are included within the article.
